# Assessment of the Psychosocial Impact of Pancreatic Cancer Surveillance in High-Risk Individuals

**DOI:** 10.3390/cancers16010086

**Published:** 2023-12-23

**Authors:** Isabel Anez-Bruzual, Sarah Coughlin, Daniel Clay, Jordan Heiman, Michaela Dungan, Marina Weber, Christopher V. Almario, Galen Leung, Nuzhat A. Ahmad, Gregory G. Ginsberg, Michael L. Kochman, Kathleen D. Valverde, Jessica M. Long, Bryson W. Katona

**Affiliations:** 1 Master of Science in Genetic Counseling Program, Perelman School of Medicine, University of Pennsylvania, Philadelphia, PA 19104, USA; isabel_anez@dfci.harvard.edu (I.A.-B.); kathleen.valverde@pennmedicine.upenn.edu (K.D.V.); 2 Division of Gastroenterology and Hepatology, Perelman School of Medicine, University of Pennsylvania, Philadelphia, PA 19104, USA; sarah.coughlin@pennmedicine.upenn.edu (S.C.); daniel.clay@pennmedicine.upenn.edu (D.C.); jheim607@gmail.com (J.H.); michaela.dungan@pennmedicine.upenn.edu (M.D.); marina.weber@pennmedicine.upenn.edu (M.W.); galen.leung@pennmedicine.upenn.edu (G.L.); nuzhat.ahmad@pennmedicine.upenn.edu (N.A.A.); gregory.ginsberg@pennmedicine.upenn.edu (G.G.G.); michael.kochman@pennmedicine.upenn.edu (M.L.K.); 3 Karsh Division of Gastroenterology and Hepatology, Cedars-Sinai Medical Center, Los Angeles, CA 90048, USA; christopher.almario@csmc.edu; 4 Division of Hematology-Oncology, Department of Medicine, Penn Medicine, Philadelphia, PA 19104, USA; jessica.long@pennmedicine.upenn.edu

**Keywords:** hereditary pancreatic cancer risk, endoscopic ultrasound, familial pancreatic cancer

## Abstract

**Simple Summary:**

This study explores the psychosocial impact of pancreatic cancer (PC) surveillance in individuals at high-risk (HRIs) of developing PC. The primary objective was to understand the attitudes and beliefs of HRIs undergoing PC surveillance and assess the immediate and sustained psychosocial effects. By investigating factors such as perceived benefits, self-efficacy, and emotions before and after surveillance, the study aims to shed light on the overall experience of PC surveillance. The findings suggest that PC surveillance can yield lasting psychosocial benefits for HRIs. This insight not only enhances our understanding of the social and psychological aspects of surveillance, but also has implications for how the medical community approaches and supports individuals in high-risk PC surveillance programs.

**Abstract:**

Objectives: Pancreatic cancer (PC) surveillance of high-risk individuals (HRIs) downstages PC and improves survival. However, it remains less clear whether PC surveillance has a positive psychosocial impact on HRIs. Herein, we aimed to define the attitudes and beliefs of HRIs undergoing PC surveillance, and the immediate and sustained psychosocial impact of PC surveillance in HRIs. Methods: 100 HRIs undergoing PC surveillance by endoscopic ultrasound (EUS) completed three surveys addressing different components of the psychosocial impact of PC surveillance. Logistic regression analyses were performed to identify predictive factors relating to these components. Results: Most HRIs reported increased perceived benefits of PC surveillance, self-efficacy, and perceived severity of PC. HRIs reported few negative emotions prior to surveillance and frequent positive emotions after surveillance. Compared to prior to surveillance, there was a 53.5% decrease in the level of distress reported by HRIs after surveillance, which was sustained for 4–6 weeks post-surveillance. Family history of PC and lower self-reported mental health were identified as predictors for increased perceived susceptibility to PC (*p* < 0.01) and greater change in distress pre- to post-surveillance (*p* < 0.01), respectively. Conclusions: Our findings suggest that PC surveillance can lead to sustained psychosocial benefits in HRIs.

## 1. Introduction

Pancreatic ductal adenocarcinoma (PC) is associated with a high mortality rate [1]. Survival depends on stage at diagnosis, with most PC cases identified at advanced stages [2]. If PC is detected at a localized stage when resection is feasible, the 5-year survival rate is 42% and is over 80% for stage 1A PCs [3]. However, for locally advanced and metastatic PC, the 5-year survival rate is 15% and 3%, respectively [1]. Development of effective surveillance programs to detect early-stage PCs may allow the opportunity for definitive multimodal therapy with prolonged survival.

Due to the relatively low incidence of PC in the general population [1,2], current guidelines support PC surveillance only for high-risk individuals (HRIs), generally defined as those with familial PC (at least two family members with PC who are directly related, one being a first-degree relative of the HRI) and/or those with an identified pathogenic germline variant (PGV) associated with PC. These PGVs include genes associated with hereditary breast and ovarian cancer (*ATM*, *BRCA1*, *BRCA2*, *PALB2*) and Lynch syndrome (*MLH1*, *MSH2/EPCAM*, *MSH6*, *PMS2*) in combination with a family history of PC, hereditary pancreatitis (*PRSS1*, *PRSS2*, *CTRC*) with a clinical history of pancreatitis, Peutz–Jeghers syndrome (*STK11*), and familial atypical multiple mole melanoma syndrome (*CDKN2A*) [4]. PC surveillance using magnetic cholangiopancreatography (MRCP) and endoscopic ultrasound (EUS) has demonstrated utility in detecting precursor lesions and PCs at earlier, resectable stages with improved overall survival [5,6,7]. While early detection is a critically important benefit, PC surveillance may also meaningfully impact HRIs’ mental health. However, there remain limited data regarding whether PC surveillance leads to psychosocial benefits for HRIs.

Psychosocial impact may be defined as the effect of an intervention (e.g., PC surveillance) on an individual’s social and/or psychological aspects [8]. While the psychosocial impact of surveillance has been extensively studied for other familial cancers, few studies evaluate the psychosocial impact of PC surveillance. Studies on surveillance for other cancers demonstrate surveillance may trigger negative emotional responses, such as worry and anxiety [9,10]. However, most participants experience decreased distress and report fewer health-related concerns after a negative surveillance exam [11,12]. Limited studies suggest annual PC surveillance generates positive psychological outcomes [13,14], but further studies are needed to evaluate extent and durability of these effects.

The psychosocial impact of PC surveillance can incorporate an HRI’s attitudes and beliefs towards PC, psychological consequences of surveillance, motivation to participate in surveillance, and surveillance-associated distress. Attitudes and beliefs regarding different cancer types have been studied using the Health Belief Model (HBM), which characterizes attitudes and behaviors as influenced by six constructs: perceived susceptibility (individual’s subjective perception of the risk of acquiring a condition), perceived severity (individual’s belief that a condition could have serious consequences), perceived benefits (individual’s belief that a particular course of action would reduce susceptibility or severity or lead to other positive outcomes), perceived barriers (individual’s feelings on the obstacles to performing a recommended health action), cues to action (the stimulus needed to trigger the decision-making process to accept a recommended health action), and self-efficacy (individual’s confidence in their ability to successfully perform a behavior) [15,16,17]. For PC in particular, the HBM has been used to predict healthy behaviors [18], such as engaging in PC surveillance, but it has not been used to assess psychosocial impact in other ways, such as evaluating cancer worry and distress. Determining whether certain attitudes or beliefs influence the psychosocial impact of surveillance, or identifying groups where the psychosocial impact may be more significant, could aid in identifying individuals considering PC surveillance who may need additional psychosocial support, including counseling and education.

In this study, we aim to define the baseline attitudes and beliefs of HRIs undergoing PC surveillance, as well as the immediate and sustained effects of PC surveillance on psychosocial factors such as motivations or distress.

## 2. Materials and Methods

A pretest-posttest survey design was utilized to assess baseline attitudes and beliefs, as well as the psychosocial impact of PC surveillance in HRIs and whether it persisted over time. All HRIs undergoing EUS for PC surveillance through Penn Medicine’s Pancreatic Cancer Risk Management Program were offered enrollment prior to their routinely scheduled surveillance EUS between April 2022 and December 2022. This study was approved by the University of Pennsylvania Institutional Review Board on 11 April 2022 (protocol number 851022).

Following verbal consent, two surveys were administered at three different time points. The first (“pretest survey”) was administered immediately before EUS in the pre-operative holding area. The second (“posttest survey”) was administered twice: (1) on the procedure day after the participant received their EUS results and (2) 4–6 weeks after the EUS procedure. The pretest and first posttest surveys occurred in person using paper surveys, whereas the second posttest survey was administered verbally by telephone by a single research study team member. The study was completed once 100 individuals had at minimum successfully completed the pretest and first posttest survey.

Survey questions were adapted from previously validated surveys studying different aspects of the psychosocial impact of cancer surveillance: attitudes and beliefs towards PC based on four of the HBM constructs (perceived susceptibility, perceived severity, perceived benefits, and self-efficacy), emotional consequences of surveillance, motivation to participate in PC surveillance (relates to the HBM’s cues to action), and distress [19,20,21,22]. Perceived barriers were not assessed given that participants were approached when presenting for surveillance, suggesting there were not any major obstacles in performing the recommended health action. The pretest survey (Supplemental Instrument S1) included 20 questions assessing attitudes and beliefs towards PC using a 5-point Likert scale (1 = “Strongly disagree” to 5 = “Strongly agree”), a multiple selection checklist evaluating motivation for undergoing PC surveillance, 5 questions assessing emotional consequences of having to undergo surveillance using a 4-point Likert scale (1 = “Not at all” to 4 = “All of the time)”, the distress thermometer [21] to assess the level of distress before surveillance, and two questions from the PROMIS Global Health Instrument [23] evaluating baseline self-ratings of physical and mental health on a 5-point Likert scale (1 = “Excellent” to 5 = “Poor”). The posttest survey (Supplemental Instrument S2) included 5 questions assessing emotional consequences of having undergone surveillance using a 4-point Likert scale (1 = “Not at all” to 4 = “All of the time), a question evaluating likelihood of continuing PC surveillance on a 5-point Likert scale (1 = “Very unlikely” to 5 = “Very likely”), and the distress thermometer to assess the levels of distress after surveillance. Prior to implementation, the survey was reviewed by a small cohort of HRIs (*n* = 5) to confirm clarity and ease of completion.

Participant demographics, personal history of cancer, genetic testing results, family history of cancer, PC surveillance history, and latest PC surveillance results were obtained from the electronic medical record and stored in a secure REDCap database along with survey responses.

Continuous variables were reported as means with standard deviation reported. All binary and categorical variables of interest were reported proportions and counts. Two sample T tests were used to compare the means or continuous variables. Pearson χ^2^ tests were used to estimate *p*-values comparing binary and categorical variables. Exploratory univariate and multivariate logistic regression analyses were additionally performed to identify factors predictive of testing change in distress following EUS. Exploratory univariate and multivariate linear regression analyses were performed to identify factors predictive of perceived susceptibility to malignancy. A *p*-value < 0.05 was considered statistically significant for these tests. All analyses were performed using Stata/IC 15.0 or RStudio statistical programs.

## 3. Results

Of 134 consecutive HRIs undergoing EUS for PC surveillance who were offered enrollment, 100 HRIs (74%) enrolled in the study (Table 1). Participants were primarily White (96%), non-Hispanic (98%), and female (71%), with a median age of 59 years. Most participants (75%) had a PGV in a gene associated with increased risk of PC, primarily *BRCA2* (39%) and *BRCA1* (15%). Forty-two participants (42%) had a prior cancer diagnosis, and seventy-six (76%) individuals had a family history of PC. Twenty-five (25%) individuals were undergoing PC surveillance for the first time. Participants and those who declined participation were comparable except for mean age at surveillance, which was 59 years for those who participated and 66 years for those who declined (*p* = 0.01; Table 1), and the percentage of HRIs with a PGV in *ATM*, which was 4% (*n* = 5) for those who participated and 14.7% (*n* = 5) for those who declined.

All 100 participants completed the pretest and first posttest survey, while the second posttest survey had a 98% completion rate. A total of 95 participants had no concerning pancreatic findings on their EUS, defined as a new pancreatic mass or cyst greater than 1 cm. Of the remaining 5, 1 had PC diagnosed, and 4 had potential solid lesions that proved benign after FNA and/or additional imaging.

**Figure 1:**
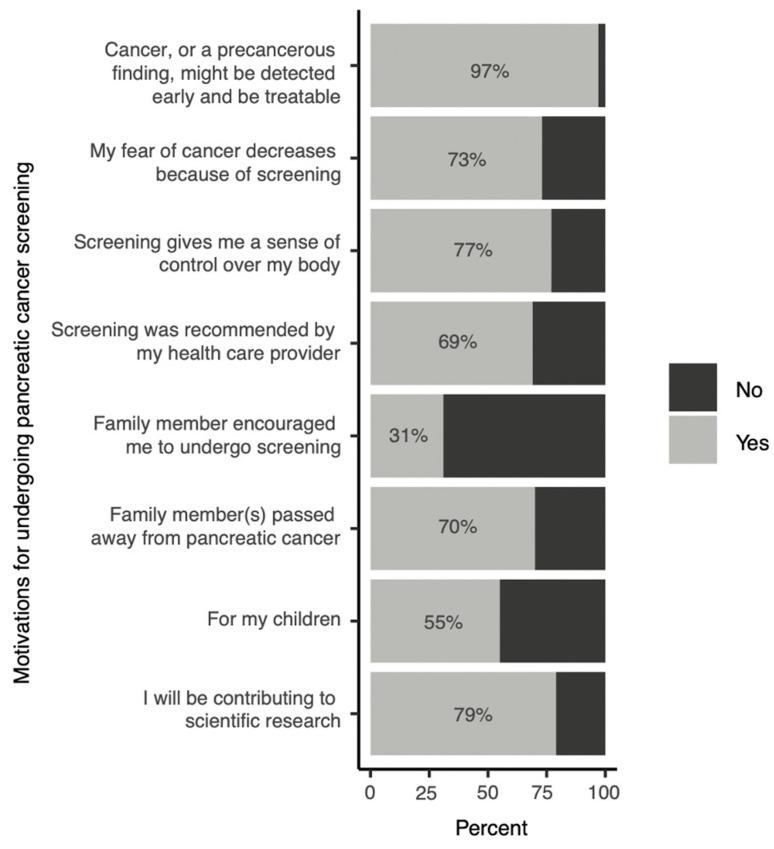
Self-reported motivations for HRIs to undergo pancreatic cancer surveillance.

In the pretest survey, 97% of HRIs identified the possibility of early detection of malignancy or a precancerous lesion as one of their motivations for undergoing PC surveillance (Figure 1). A majority of individuals (55–79%) also identified the following motivations: reduced fear of PC following surveillance, gaining a sense of control over their bodies, a health care provider’s recommendation, a family member passing away from PC, their children, and contributing to scientific research. Only 33% reported a family member’s encouragement as a motivation (Figure 1).

**Figure 2:**
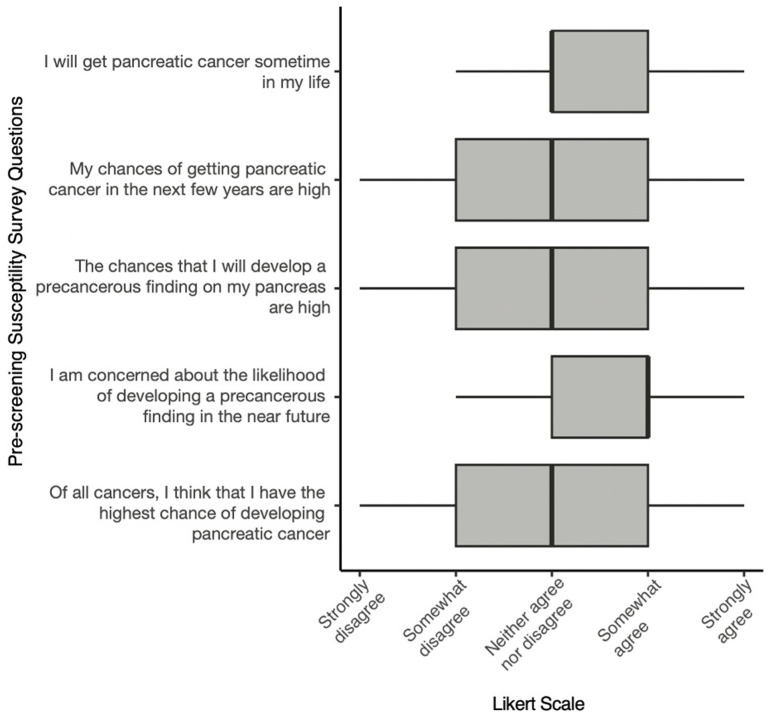
Distribution of responses to questions assessing perceived susceptibility to PC or precancerous lesions in the pancreas.

Most HRIs demonstrated increased self-efficacy, perceived severity of PC, and perceived benefits of PC surveillance (Supplemental Figure S1). Responses also indicated a low level of concern regarding risks or discomfort associated with EUS. Amongst the HBM constructs, perceived susceptibility generated the largest response variability (Figure 2). Univariate and multivariate linear regression analyses revealed a family history of PC as the strongest predictor of increased perceived susceptibility to PC (*p* < 0.01) (Supplemental Table S1). Black race appeared to correlate with lower levels of perceived susceptibility based on the multivariate regression analysis (*p* = 0.03) (Supplemental Table S1), but there were only 3 individuals in the cohort that identified as Black and therefore these results should be interpreted with caution. There were no significant differences in the levels of perceived susceptibility of HRIs based on age, sex, whether the participant carried a PGV, or whether this was the participant’s first surveillance exam (Supplemental Table S1).

**Figure 3:**
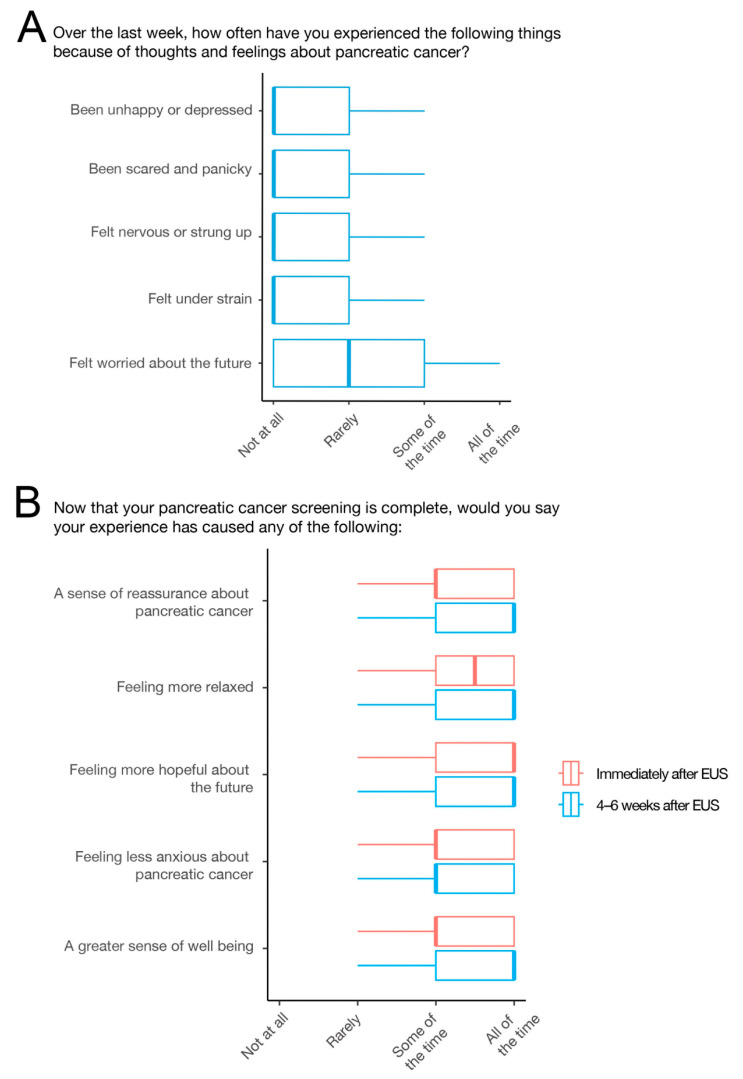
Emotional consequences of PC surveillance. (**A**) Distribution of responses assessing negative emotional effects of having to undergo surveillance (green). (**B**) Distribution of positive emotional effects immediately after surveillance (red) and 4–6 weeks after surveillance (blue).

In the 7 days prior to surveillance, HRIs reported infrequent negative emotions such as unhappiness or depression, fear or panic, nervousness, stress, or worry about the future (Figure 3A). Immediately after EUS completion, individuals reported feelings of reassurance, relaxation, hopefulness about the future, reduced anxiety about PC, and a greater sense of wellbeing (Figure 3B). These positive emotions persisted at 4–6 weeks post-EUS (Figure 3B).

**Figure 4:**
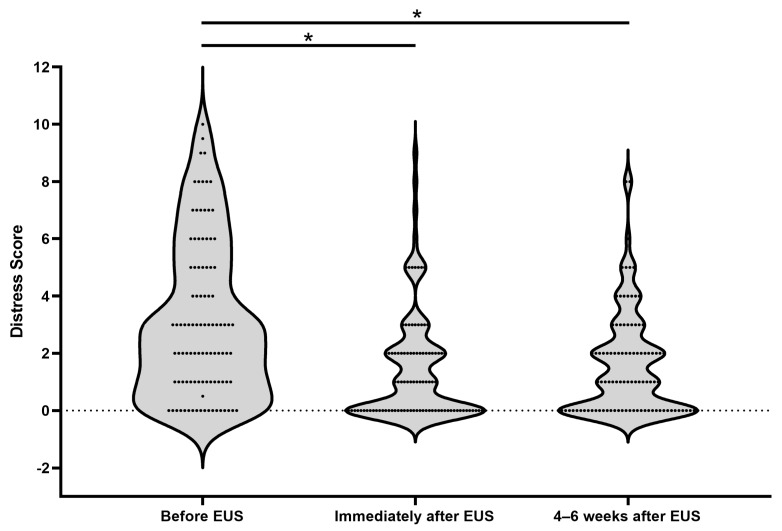
Distribution of distress levels reported before, immediately after, and 4–6 weeks after EUS. Mean distress score before EUS = 3.14 (SEM 0.27, 95% CI 2.60–3.68), immediately after EUS = 1.46 (SEM 0.19, 95% CI 1.08–1.84), and 4–6 weeks after EUS = 1.57 (SEM 0.18, 95% CI 1.21–1.93). * *p* < 0.01.

Compared to pre-EUS distress levels, there was a 53.5% decrease in the level of distress reported by HRIs following receipt of EUS results (*p* < 0.01; Figure 4). This reduction was sustained at 4–6 weeks post-EUS with a 50% overall decrease compared to pre-EUS (*p* < 0.01; Figure 4). Univariate linear regression analysis supports a higher pre-EUS PROMIS mental health scale score (consistent with worse self-reported mental health) as a predictor of greater change in reported distress pre- and post-EUS (*p* < 0.01; Table 2). Multivariate linear regression analysis (*p* = 0.04; Table 2) shows that the same relationship holds after adjusting for demographic variables, prior screening experience, personal and family history of cancer, and diagnosis of a PGV. In contrast, female sex was associated with increased change in distress by univariate analysis (*p* = 0.02), but not after adjusting for other demographic and clinical factors by multivariate analysis (*p* = 0.05). There were no significant differences in distress levels by age, race/ethnicity, whether the participant carried a PGV, whether this was the participant’s first surveillance exam, or family history of PC.

## 4. Discussion

PC surveillance in HRIs is important for early detection of PC and has been shown to downstage PC at diagnosis and improve long-term survival. However, there may be other advantages of PC surveillance in HRI, namely psychosocial benefits, which we assessed in this study of HRIs undergoing PC surveillance through both pre- and post-surveillance surveys. The psychosocial assessment administered focused on four factors pertaining to the psychosocial impact of PC surveillance including attitudes and beliefs about PC and PC surveillance, motivations for surveillance, emotional consequences of surveillance, and surveillance-related distress, with the overall study results supporting that PC surveillance provides important psychosocial benefit to HRIs.

Increased perceived susceptibility has been identified as a predictor of preventative health behaviors, including continued PC surveillance [24,25]. Thus, increased perceived susceptibility appears to be another important motivator among those participating in PC surveillance, along with the possibility of early detection of malignancy or a precancerous lesion, contributing to scientific research, reduced fear of cancer following surveillance, family history of PC, healthcare provider’s recommendation, and participants’ children. Understanding HRIs’ motivations for participating in surveillance could be helpful in improving PC surveillance uptake and the shared decision-making process. Clinician recommendations have been identified as a consistent predictor of participation in surveillance programs for other cancers [26,27,28]. Therefore, by identifying various factors motivating HRIs to engage in PC surveillance, providers can tailor communication and education strategies to address these factors and potentially improve adherence to the surveillance program.

This study supports the psychological benefits of surveillance. HRIs reported few negative emotions in response to thoughts about PC in the week leading up to EUS and frequent positive emotions after EUS, as well as statistically significantly reduced distress levels after their surveillance EUS. Importantly, these positive changes persisted 4–6 weeks after surveillance, suggesting PC surveillance has a positive and enduring effect on HRIs. Negative emotions as a consequence of surveillance for other cancers have been associated with lower rates of participation in surveillance programs [29,30]. In a setting where surveillance is associated with positive psychological consequences, HRIs may be more likely to continue participating in the surveillance program. These findings highlight the importance of considering emotional well-being when designing and implementing PC surveillance programs, as it pertains to the feasibility of such programs. By recognizing and highlighting the psychological benefits of PC surveillance, healthcare providers may encourage long term commitment to PC surveillance, resulting in better health outcomes of HRIs.

While there was an overall decrease in distress post-EUS in this study population, this finding was driven in part by a subset of individuals who reported lower mental health scores and higher levels of distress before EUS, which were ameliorated after surveillance. Increases in distress specific to cancer risk has been identified as a psychological factor affecting surveillance adherence [31]. This finding highlights the importance of identifying HRIs with lower mental health scores, who may benefit from additional counseling about the potential benefits of surveillance. Clinicians might proactively provide support resources to ameliorate surveillance-associated stress, such as counseling, stress management techniques, or referrals to mental health specialists. By providing additional support to these individuals, healthcare providers can help mitigate the negative impact of distress and improve the overall wellbeing of patients.

Overall, HRIs electing to pursue EUS expressed increased perceived benefits of PC surveillance, self-efficacy, and perceived severity of PC. In addition, individuals with family history of PC reported greater perceived susceptibility to PC compared to individuals without family history of PC. These findings are consistent with previous studies denoting family history of PC as a predictor of increased perceived PC lifetime risk [32,33]. This relationship between increased perceived susceptibility and having a family history has also been reported for other cancers [34,35]. This study augments these findings by documenting the relationship between heightened PC risk perception and family history in a cohort of HRIs undergoing PC surveillance.

Despite significant findings, several limitations should be considered when interpreting study results. First, the study enrolled HRIs from a single site with the majority of participants representing a single demographic (non-Hispanic White females). In addition, there was no control group, resulting in a lack of corresponding data as a comparator. Therefore, the findings of this study might not be generalizable to a more diverse group. However, this is a pervasive issue in many PC surveillance-focused studies where there is limited racial, ethnic, and sex-based diversity amongst individuals undergoing surveillance [32,36]. Second, survey questions were self-adapted from previously validated questionnaires. While the questions were only modified to specifically address PC and PC surveillance, this could have introduced some bias. Lastly, analysis was limited to a 4–6-week follow-up period post-EUS, a relatively short time frame.

To address the limitations identified in this study, several strategic approaches can be considered for future studies. First and foremost, future research should prioritize multi-site recruitment with diverse participant demographics, ensuring a more representative sample. Such an effort would need to involve collaboration with multiple institutions and the inclusion of targeted recruitment strategies to increase the participation of underrepresented groups in PC early detection programs. Additionally, longitudinal studies with extended follow-up periods beyond 4–6 weeks post-EUS are needed to determine the extent of the duration of benefits of PC surveillance. These potential future approaches would improve the generalizability of the study’s outcomes, ultimately advancing our knowledge in the field of PC surveillance.

This study provides valuable insights into the potential psychosocial benefits of EUS for PC surveillance, which could inform the design and implementation of future studies. Future research could explore potential benefits of incorporating mental health interventions, such as counseling or stress management techniques, into PC surveillance programs to help those with elevated distress better appreciate the benefits of surveillance. Additionally, through providing support to patients, healthcare providers might augment adherence to PC surveillance programs and ultimately reduce morbidity and mortality from PC in HRIs. Finally, understanding the psychosocial impact of PC surveillance is critical for improving the overall care of HRIs and for developing more effective screening programs addressing both the medical and psychological needs of patients.

## Figures and Tables

**Table 1:** Cohort characteristics (Abbreviations: SD, standard deviation; PGV, pathogenic variant; PC, pancreatic cancer).

	Participated in Survey	Declined Survey Participation	*p*-Value
	*n* = 100	*n* = 34	
**Age at surveillance (mean, SD)**	59.0 (53.5–64.5)	66.0 (57.0–71.0)	0.01
**Sex**			0.34
Male	29.0% (29)	20.6% (7)	
Female	71.0% (71)	79.4% (27)	
**Race**			0.50
White	96.0% (96)	100.0% (34)	
Black	3.0% (3)	0.0% (0)	
Asian	1.0% (1)	0.0% (0)	
**Ethnicity**			0.41
Hispanic or Latino	2.0% (2)	0.0% (0)	
Not Hispanic or Latino	98.0% (98)	100.0% (34)	
**Personal history of cancer**	42.0% (42)	47.1% (16)	0.61
**Family history of pancreatic cancer**	76.0% (76)	64.7% (22)	0.20
**Prior genetic testing**	96.0% (96)	87.9% (29)	0.09
**Presence of a PGV in a PC risk gene**	75.0% (72)	79.3% (23)	0.63
*ATM*	4.0% (4)	14.7% (5)	0.03
*BRCA1*	15.0% (15)	11.8% (4)	0.64
*BRCA2*	39.0% (39)	35.3% (12)	0.70
*CDKN2A*	1.0% (1)	2.9% (1)	0.42
*PALB2*	6.0% (6)	2.9% (1)	0.49
*STK11*	2.0% (2)	0.0% (0)	0.41
Lynch syndrome-associated PGV	6.0% (6)	2.9% (1)	0.49
Other	1% (1)	5.9% (2)	0.10
**Subject undergoing their baseline pancreatic cancer surveillance study**	25.0% (25)	26.5% (9)	0.86

**Table 2:** Linear regression analyses of factors predicting change in distress following EUS. * Compared to individuals identified as White. ** Compared to individuals identified as Hispanic or Latino.

	Univariate Linear Regression Analysis			Multivariate Linear Regression Analysis		
	Coefficient	*p*-Value	[95% Confidence Interval]	Coefficient	*p*-Value	[95% Confidence Interval]
**Change in Distress**						
Age	−0.03	0.27	−0.08 to 0.02	−0.01	0.66	−0.07 to 0.04
Female sex	1.22	0.02	0.2 to 2.22	1.10	0.05	0.01 to 2.19
*Race*						
Black *	−1.06	0.44	−3.80 to 1.68	−1.23	0.38	−4.04 to 1.57
Asian *	−1.73	0.47	−6.42 to 2.97	−1.71	0.49	−6.63 to 3.20
*Ethnicity*						
Not Hispanic or Latino **	−1.35	0.42	−4.67 to 1.98	−0.72	0.68	−4.25 to 2.80
Personal history of cancer	0.22	0.64	−0.72 to 1.17	−0.07	0.90	−1.10 to 0.97
Pathogenic gene variant	0.07	0.90	−1.05 to 1.19	−0.05	0.94	−1.27 to 1.18
First surveillance study	0.48	0.38	−0.59 to 1.55	0.54	0.36	−0.62 to 1.71
Family history of pancreatic cancer	0.40	0.47	−0.69 to 1.49	0.62	0.33	−0.64 to 1.87
Reported harm to mental health	0.71	<0.01	0.23 to 1.18	0.57	0.04	0.03 to 1.11

## Data Availability

The data presented in this study are available in this article as well as in the Supplementary Materials.

## Supplementary Materials

The following supporting information can be downloaded at: https://www.mdpi.com/article/10.3390/cancers16010086/s1, Supplemental Figure S1: Distribution of responses to questions assessing self-efficacy, perceived severity of PC and PC surveillance, and perceived benefits of PC surveillance; Supplemental Table S1: Linear regression analyses of factors predicting perceived susceptibility to malignancy; Supplemental Instrument S1: Pretest survey; Supplemental Instrument S2: Posttest survey.
