# Supplementary material for: Assessment of the Psychosocial Impact of Pancreatic Cancer Surveillance in High-Risk Individuals
Source: Cancers (Basel). 2023 Dec 23;16(1):86. doi: 10.3390/cancers16010086 (PMC10777978; doi:10.3390/cancers16010086)

**Supplemental Figure S1.** Distribution of responses to questions assessing self-efficacy, perceived severity of PC and PC surveillance, and perceived benefits of PC surveillance.

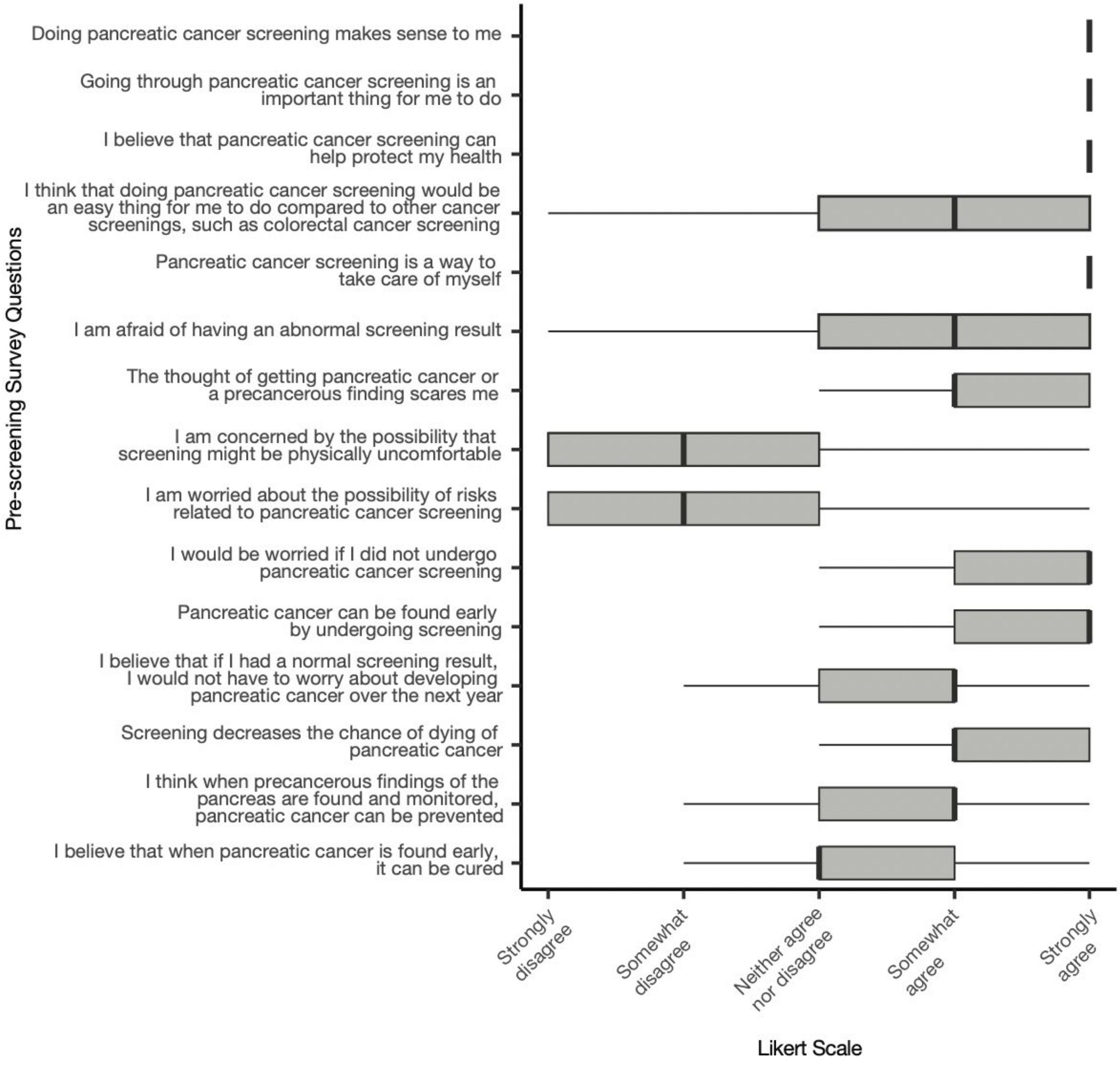

**Supplemental Table S1.** Linear regression analyses of factors predicting perceived susceptibility to malignancy. *Compared to individuals identified as White. **Compared to individuals identified as Hispanic or Latino.

| | Univariate Linear Regression Analysis | | | Multivariate Linear Regression Analysis | | |
|---|---|---|---|---|---|---|
| | **Coefficient** | **P-value** | **[95% Confidence Interval]** | **Coefficient** | **P-value** | **[95% Confidence Interval]** |
| **Perceived susceptibility to malignancy** | | | | | | |
| Age | <0.01 | 0.67 | -0.02 to 0.02 | <0.01 | 0.89 | -0.02 to 0.02 |
| Female sex | -0.14 | 0.48 | -0.52 to 0.24 | -0.16 | 0.38 | -0.52 to 0.20 |
| Race | | | | | | |
| Black* | -0.85 | 0.10 | -1.86 to 0.16 | -1.01 | 0.03 | -1.93 to -0.08 |
| Asian* | 0.02 | 0.99 | -1.71 to 1.74 | -0.20 | 0.80 | -1.81 to 1.40 |
| Ethnicity | | | | | | |
| Not Hispanic or Latino** | 0.37 | 0.56 | -0.87 to 1.60 | -0.58 | 0.32 | -1.74 to 0.58 |
| Personal history of cancer | -0.29 | 0.10 | -0.63 to 0.06 | -0.28 | 0.11 | -0.63 to 0.06 |
| Pathogenic gene variant | -0.43 | 0.04 | -0.82 to -0.03 | -0.05 | 0.82 | -0.45 to 0.36 |
| First surveillance study | -0.2 | 0.32 | -0.60 to 0.20 | <0.01 | 0.99 | -0.39 to 0.38 |
| Family history of pancreatic cancer | 0.78 | <0.01 | 0.40 to 1.15 | 0.917 | <0.01 | 0.50 to 1.33 |

**Supplemental Instrument S1.** Pretest survey

How much do you agree or disagree with each of the following statements? [Circle the option that best describes how you feel]

| | *Strongly disagree* | *Somewhat disagree* | *Neither agree nor disagree* | *Somewhat agree* | *Strongly agree* |
|---|---|---|---|---|---|
| 1. I will get pancreatic cancer sometime in my life. | 1 | 2 | 3 | 4 | 5 |
| 2. My chances of getting pancreatic cancer in the next few years are high. | 1 | 2 | 3 | 4 | 5 |
| 3. The chances that I will develop a precancerous finding on my pancreas are high. | 1 | 2 | 3 | 4 | 5 |
| 4. I am concerned about the likelihood of developing a precancerous finding in the near future. | 1 | 2 | 3 | 4 | 5 |
| 5. Of all cancers, I think that I have the highest chance of developing pancreatic cancer. | 1 | 2 | 3 | 4 | 5 |
| 6. Doing pancreatic cancer screening makes sense to me. | 1 | 2 | 3 | 4 | 5 |
| 7. Going through pancreatic cancer screening is an important thing for me to do. | 1 | 2 | 3 | 4 | 5 |
| 8. I believe that pancreatic cancer screening can help protect my health. | 1 | 2 | 3 | 4 | 5 |

| | *Strongly disagree* | *Somewhat disagree* | *Neither agree nor disagree* | *Somewhat agree* | *Strongly agree* |
|---|---|---|---|---|---|
| 9. I think that doing pancreatic cancer screening would be an easy thing for me to do compared to other cancer screenings, such as colorectal cancer screening. | 1 | 2 | 3 | 4 | 5 |
| 10. Pancreatic cancer screening is a way to take care of myself. | 1 | 2 | 3 | 4 | 5 |
| 11. I am afraid of having an abnormal screening result. | 1 | 2 | 3 | 4 | 5 |
| 12. The thought of getting pancreatic cancer or a precancerous finding scares me. | 1 | 2 | 3 | 4 | 5 |
| 13. I am concerned by the possibility that screening might be physically uncomfortable. | 1 | 2 | 3 | 4 | 5 |
| 14. I am worried about the possibility of risks related to pancreatic cancer screening. | 1 | 2 | 3 | 4 | 5 |
| 15. I would be worried if I did not undergo pancreatic cancer screening | 1 | 2 | 3 | 4 | 5 |
| 16. Pancreatic cancer can be found early by undergoing screening. | 1 | 2 | 3 | 4 | 5 |
| 17. I believe that if I had a normal screening result, I would not have to worry about developing pancreatic cancer over the next year. | 1 | 2 | 3 | 4 | 5 |

| | *Strongly disagree* | *Somewhat disagree* | *Neither agree nor disagree* | *Somewhat agree* | *Strongly agree* |
|---|---|---|---|---|---|
| 18. Screening decreases the chance of dying of pancreatic cancer. | 1 | 2 | 3 | 4 | 5 |
| 19. I think when precancerous findings of the pancreas are found and monitored, pancreatic cancer can be prevented. | 1 | 2 | 3 | 4 | 5 |
| 20. I believe that when pancreatic cancer is found early, it can be cured. | 1 | 2 | 3 | 4 | 5 |

My motive(s) for undergoing pancreatic cancer screening include: [Check all that apply]

☐ Cancer, or a precancerous finding, might be detected early and be treatable.
☐ My fear of cancer decreases because of screening.
☐ Screening gives me a sense of control over my body.
☐ Screening was recommended by my health care provider.
☐ Family member encouraged me to undergo screening.
☐ Family member(s) passed away from pancreatic cancer.
☐ For my children.
☐ I will be contributing to scientific research.
☐ Other (please explain):

______________________________________________

Over the last week, how often have you experienced the following things because of thoughts and feelings about pancreatic cancer? [Circle the option that best describes how you feel]

| | *Not at all* | *Rarely* | *Some of the time* | *All of the time* |
|---|---|---|---|---|
| 1. Been unhappy or depressed | 1 | 2 | 3 | 4 |
| 2. Been scared and panicky | 1 | 2 | 3 | 4 |
| 3. Felt nervous or strung up | 1 | 2 | 3 | 4 |
| 4. Felt under strain | 1 | 2 | 3 | 4 |
| 5. Felt worried about the future | 1 | 2 | 3 | 4 |

Distress is an unpleasant experience of mental, physical, social, or spiritual nature. It can affect the way you think, feel, or act. Please circle the number (0–10) that best describes how much distress you have been experiencing in the past week, including today.

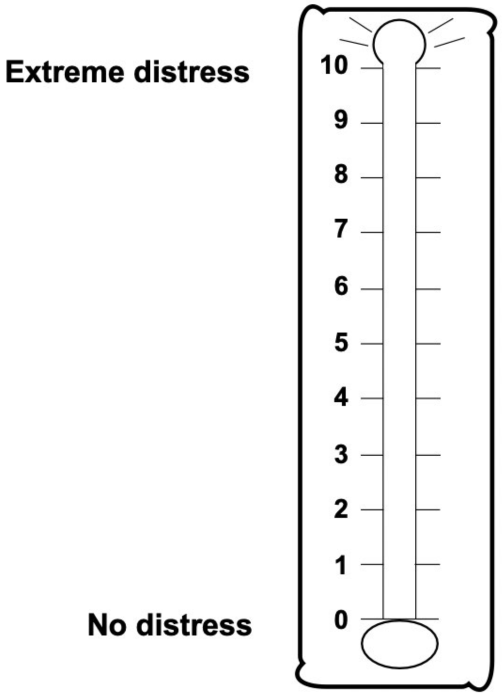

In general, how would you rate your physical health? [Circle the option that applies best]

(1) Excellent
(2) Very good
(3) Good
(4) Fair
(5) Poor

In general, how would you rate your mental health, including your mood and your ability to think? [Circle the option that applies best]

(1) Excellent
(2) Very good
(3) Good
(4) Fair
(5) Poor

**Supplemental Instrument S2.** Posttest survey

Now that your pancreatic cancer screening is complete, would you say your experience has caused any of the following:

| | *Not at all* | *Rarely* | *Some of the time* | *All of the time* |
|---|---|---|---|---|
| 1. A sense of reassurance about pancreatic cancer | 1 | 2 | 3 | 4 |
| 2. Feeling more relaxed | 1 | 2 | 3 | 4 |
| 3. Feeling more hopeful about the future | 1 | 2 | 3 | 4 |
| 4. Feeling less anxious about pancreatic cancer | 1 | 2 | 3 | 4 |
| 5. A greater sense of well being | 1 | 2 | 3 | 4 |

How likely is it that you will continue pancreatic screening in the future? [Circle the one that applies best]

(a) Very unlikely
(b) Somewhat unlikely
(c) Neither likely nor unlikely
(d) Somewhat likely
(e) Very likely

Distress is an unpleasant experience of mental, physical, social, or spiritual nature. It can affect the way you think, feel, or act. Please circle the number (0–10) that best describes how much distress you are feeling after completing pancreatic cancer screening.

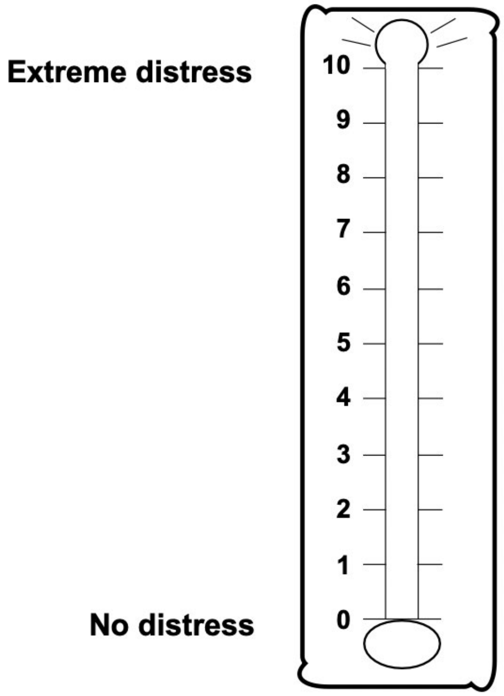

Supplement: Supplementary file 1 [file cancers-16-00086-s001.zip › cancers-2690488-supplementary.pdf]
